# STEP-COVID: a pilot study of a prenatal intervention for pregnant women during the COVID-19 pandemic

**DOI:** 10.1038/s41598-023-33369-8

**Published:** 2023-04-20

**Authors:** Nicolas Berthelot, Julia Garon-Bissonnette, Christine Drouin-Maziade, Vanessa Bergeron, Thibaut Sériès

**Affiliations:** 1 Department of Nursing Sciences, Université du Québec à Trois-Rivières, Trois-Rivières, Quebec Canada; 2 Department of Psychology, Université du Québec à Trois-Rivières, Trois-Rivières, Quebec Canada; 3 Centre d’études interdisciplinaires sur le développement de l’enfant et la famille (CEIDEF), Trois-Rivières, Québec Canada; 4 CERVO Brain Research Center, Quebec City, Quebec Canada; 5 Interdisciplinary Research Center on Intimate Relationship Problems and Sexual Abuse (CRIPCAS), Montreal, Quebec Canada; 6 Groupe de recherche et d’intervention auprès des enfants vulnérables et négligés (GRIN), Trois-Rivières, Quebec Canada

**Keywords:** Psychology, Risk factors, Preventive medicine

## Abstract

The COVID-19 pandemic has been associated with a global increase in psychological distress in pregnant women. This study evaluated the effects of STEP-COVID, a six-session mentalization-based prenatal group program offered online during the COVID-19 pandemic. The 100 participants were allocated to STEP-COVID or to the natural trajectory of prenatal care. Pre- and post-intervention assessments included measures of psychological distress, post-traumatic symptoms and positive affectivity. Perception of change during pregnancy on resilience-promoting factors was also assessed at post-intervention. A significant decrease in psychological distress and post-traumatic symptoms and an increase in positive affectivity were observed in participants in the intervention condition, whereas only post-traumatic symptoms improved in the control condition. Women who participated in STEP-COVID also reported greater changes during pregnancy on resilience-promoting factors than women in the control condition. Results hold promise for buffering the effect of the pandemic on the mental health of pregnant women using brief online interventions. Clinical trial registration: NCT05419167 (15/06/2022)

## Introduction

During the first two years of the COVID-19 pandemic, a dozen systematic reviews and meta-analyses, including overall more than 115 empirical studies, reported an increase in psychological distress in pregnant women worldwide^1–10^. This enormous level of interest in the mental health of pregnant women during the pandemic was somewhat predictable given the well documented adverse effects of prenatal stress on maternal functioning and on offspring development^11–14^. Recent studies have confirmed that the psychological distress provoked by the pandemic in pregnant women would similarly impact maternal functioning and offspring early development^15,16^. As a response to this situation, many scholars, clinicians and experts published “calls to action” advocating that pilot research evaluating behavioral interventions should be prioritized to buffer the effects of the COVID-19 pandemic on pregnant women’s mental health^17–21^. Yet, intervention studies remain scarce, if not nonexistent.

### STEP-COVID: a prenatal group intervention

To respond to the upsurge in psychological distress in pregnant women during the pandemic and to do so while conforming to the restrictions in place so as to limit the propagation of the virus (e.g., social distancing), we adapted a mentalization-based and trauma-informed group program initially developed for pregnant women with histories of childhood traumas called the STEP program^22–24^, with the purpose to reach all pregnant women, whether or not they experienced traumas during their childhood. The resulting adaptation (STEP-COVID: Supporting the Transition to and Engagement in Parenthood during the COVID-19 pandemic) is a 6-week group intervention offered online by two facilitators (including at least one psychologist or another professional with significant experience in mental health and mentalization-based interventions). Sessions last two hours and are offered in a synchronous mode to groups of four to six pregnant women. In line with Lassri and Desatnik^25^ and Penner and Rutherford’s^19^ remarks that improving mentalization and emotion regulation during pregnancy would have multiple positive outcomes for mothers, their infant, and the mother-infant relationship in times of heightened stress such as the COVID-19 pandemic, the general goals of the STEP-COVID program are to foster emotion regulation and reflective capacities. More precisely, the program aims to (a) support mentalization in relation to oneself, motherhood and the relationship with the child to be born, (b) reduce isolation by allowing participants to exchange about the positive aspects and the challenges of pregnancy and motherhood with other women, (c) explore what pregnant women are going through in the context of the pandemic, (d) allow participants to repossess their experience of pregnancy during this period of insecurity and fear, and (e) consider new ways of coping with stress and unpleasant emotions. The program is manualized and uses structured activities based on theoretical grounds and empirical research during which facilitators share information, animate reflective activities, and facilitate exchanges.

The intervention is divided into two phases, each including three sessions. The first three sessions aim to explore how the participants feel (making sure to pay attention to both pleasant and unpleasant emotions), to better understand what makes them feel this way, to allow them to exchange with other people who are going through similar experiences, and to support the ability to manage stress and more unpleasant emotions in order to find or maintain a sense of balance. The following three sessions aim to enable participants to refocus on their experience of pregnancy and motherhood by giving them the opportunity to reflect upon how they wish to be as mothers, upon how their personal history influences their experience of pregnancy and motherhood, upon the moments that, as mothers, might be the most pleasant and those that will require more adaptations, and upon identifying the needs they have or expect to have after childbirth as well as the resources available to them to meet these needs. The intervention is inspired by mentalization-based practices and invites participants to reflect upon the thoughts and emotions underlying their behavior and to develop a similar aptitude with regard to their child to be born.

The objective of the current study was to evaluate whether STEP-COVID could contribute to mitigating psychological distress and post-traumatic symptoms, increase positive affectivity, and contribute to positive changes in self-perception, relationships with significant others, and resilience (defined as perceiving oneself as being competent in the face of challenging life circumstances).

## Methods

### Recruitment strategy

Participants were recruited through advertisements at pregnancy-related medical appointment centers or on social media during the COVID-19 pandemic between September 2020 and May 2021. Participants who expressed interest in learning more about the study were contacted by phone or email by a research assistant who explained the research protocol and briefly presented the interventions. Two versions of the intervention were simultaneously offered by the research team: the original STEP program (i.e., eight to nine intervention sessions developed for pregnant women who experienced childhood traumas) and STEP-COVID. The first stage of the study consisted in completing a series of questionnaires online on a secure platform to collect *baseline data* for the evaluation of the program and to assess the eligibility criteria. Most measures were re-administered toward the end of pregnancy. The study received ethical approval from our University Ethics Committee (CER-16-226-10) and from the Institutional Review Board of our regional health center (CER-2016-016). All experiments were performed in accordance with relevant guidelines and regulations. The clinical trial is registered under number NCT05419167 (15/06/2022).

### Group assignation

#### Intervention arm

**Figure 1:**
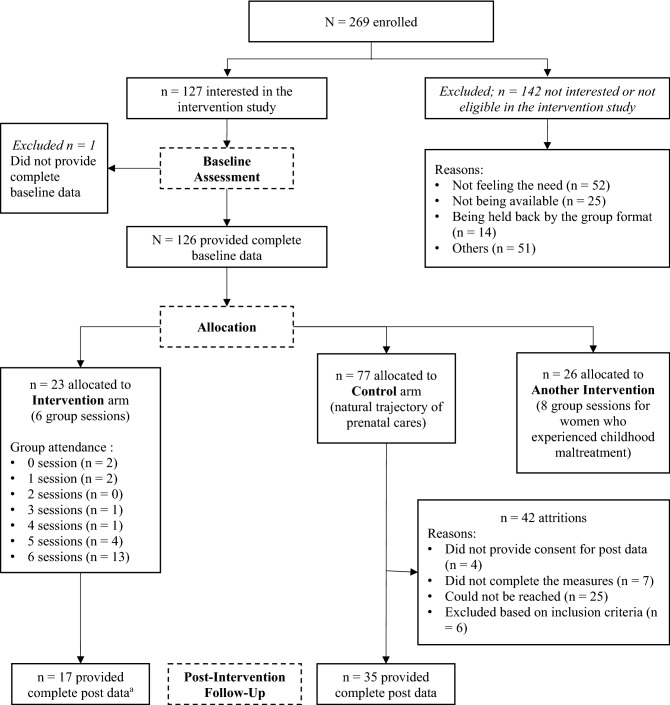
Study Flow Chart. ^a^The four participants who left the intervention before or after the first meeting had scheduling conflicts. One participant left after three sessions due to a lack of commitment in the intervention frame (e.g. not opening the camera; arriving late).

The study used a non-randomized clinical trial. Women who met the eligibility criteria based on baseline assessments (i.e., being between 12 and 28 weeks of pregnancy and being available when the program was scheduled) were invited to participate in the program. Since having experienced childhood traumas was not an exclusion criterion for participating in STEP-COVID, participants who reported having been exposed to childhood traumas at baseline assessment were invited to participate in the original program especially develop for trauma-exposed women, but were ultimately free to choose the version of the program they wished to participate in. Women interested in the STEP-COVID Program underwent a brief interview on a secure video teleconferencing platform during which the program was introduced, the conditions for participation were clarified (i.e., being able to ensure confidentiality during the meetings) and further exclusion criteria were assessed (i.e., presenting difficulties that compromise the emotional and reflective availability required by the program such as suicidal ideation, active violence, mental health disorders not stabilized, significant drug or alcohol use, self-destructive behavior, not being convinced of carrying the pregnancy to term, or experiencing a high-risk pregnancy). Twenty-three pregnant women were allocated to the intervention arm of the study (see Fig. 1).

#### Control arm

Women who could not participate in the intervention, because no group was offered at that moment or because they were unavailable when groups were scheduled, were assigned to the control arm of the study. These women did not engage in any of the STEP programs and received the regular trajectory of prenatal care (e.g., prenatal classes). Seventy-seven pregnant women were allocated to the control arm of the study (see Fig. 1). They completed the same baseline and post-intervention assessments as participants in the intervention arm at the same moment of pregnancy (see Table 1).

**Table 1:** Demographic and clinical characteristics of all women enrolled in the study who completed baseline data (N = 268).

Demographics	Included participants (n = 126)^a^	Excluded participants (n = 142)^b^	Group differences (*p* value)^c^
Age, mean (SD)	29.09 (4.92)	29.32 (4.49)	0.69
Primiparous, n (%)	90 (72.0%)	76 (53.5%)	0.002
Marital status, n (%)			
In relationship	122 (96.8%)	138 (97.2%)	0.93
Single	4 (3.2%)	4 (2.8%)	
Education level, n (%)			
High school diploma or less	11 (8.7%)	18 (12.7%)	0.80
Collegial or professional training	61 (48.4%)	59 (41.5%)	
University degree	54 (42.9%)	65 (45.8%)	
Ethnicity, n (%)			
White	119 (94.4%)	130 (91.5%)	0.22
Minority	7 (5.6%)	12 (8.5%)	
Annual household income, n (%)^d^			
Can $34,999 or less	11 (9.0%)	16 (11.7%)	0.39
Can $35,000–64,999$	22 (18.0%)	17 (12.4%)	
Can $65,000$–94,999$	41 (33.6%)	44 (32.1%)	
Can $95,000 or more	48 (39.4%)	60 (43.8%)	
Childhood trauma, n (%)	47 (38.2%)	44 (32.1%)	0.30
Mental health, mean (SD)			
Psychological distress	18.68 (5.61)	18.42 (6.52)	0.73
Post-traumatic stress symptoms	13.96 (13.3)	10.58 (10.76)	0.03
Positive affects	33.62 (5.12)	33.50 (6.14)	0.87

^a^Participants were included in the study if they showed some interest in the intervention.

^b^Participants were excluded from the present study when they refused to partake in the intervention protocol but accepted to complete research assessments.

^c^Two-sided *p*-values were obtained from *t*-tests for continuous variables and Chi-square tests for categorical variables.

^d^Nine participants did not report annual household income and eight participants did not complete baseline assessment of childhood trauma (missing data).

### Measures

#### Demographic and historical variables

Women self-reported on age, parity, marital status, education level, race/ethnicity, and annual household income at the baseline assessment. They also completed the Childhood Trauma Questionnaire (CTQ)^26^. The CTQ includes 28 items rated on a 5-point Likert scale from 1 (*never true*) to 5 (*very often true*) and assesses five types of traumas with validated cut-offs^27^. Participants with at least one subscale above the cut-off were classified as having been exposed to childhood trauma. The CTQ shows good validity across clinical and general populations^26^. In this study, Cronbach’s alpha is 0.82.

#### Evolution of mental health symptoms

Maternal mental health symptoms at baseline and post-intervention time points were assessed using three questionnaires. First, the 10-item Kessler Psychological Distress Scale (K10) assessed psychological distress using a 5-point Likert scale from 1 (*none of the time*) to 5 (*all of the time*)^28,29^. Higher scores indicate greater distress. The K10 has adequate sensitivity and specificity for the screening of mood and anxiety disorders in pregnant women^30^. In this study, Cronbach’s alpha is 0.91 (at both baseline and post-intervention).

Second, given that the COVID-19 pandemic may represent, for many, a form of trauma due to its threatening, unpredictable, extreme, and prolonged nature^31,32^, and since an increase in symptoms of post-traumatic stress disorder (PTSD) was observed in pregnant women during the pandemic^33,34^, the evolution of post-traumatic symptoms before and after the intervention was assessed using the PTSD Checklist for DSM-V (PCL-5)^35,36^. Its 20 items are rated using a 5-point Likert scale from 0 (*not at all*) to 5 (*always*). Higher scores indicate more symptoms. Both the French and original English versions have equally adequate validity and reliability^35,37^, and the instrument has been often administered to pregnant women^38,39^. In this study, Cronbach’s alpha are 0.92 (baseline) and 0.91 (post-intervention).

Finally, positive affectivity was assessed using the Positive Affect subscale of the Positive and Negative Affect Scale (PANAS)^40,41^. The 10 items of the positive affect scale are rated on a 5-point Likert scale from 1 (*very slightly or not at all*) to 5 (*extremely*). Higher scores indicate more positive affectivity such as enthusiasm, energy and dynamism. Both the French and original versions show good psychometric properties^40,41^. In this study, Cronbach’s alpha are 0.82 (baseline) and 0.85 (post-intervention).

### Perception of change during pregnancy on resilience-promoting factors

Perception of change during pregnancy was assessed at the post-intervention follow-up using two questionnaires. First, positive changes in the aftermath of stressful events were measured using the Post Traumatic Growth Inventory (PTGI)^42,43^. Its 21 items are rated on a 6-point Likert scale, from 0 (*I did not experience this change*) to 5 (*I experienced this change to a very great degree*). In this study, participants were asked to complete the questionnaire regarding changes since the beginning of pregnancy. The PTGI has five subscales reflecting different constructs: New Possibilities, Relating to Others, Personal Strength, Appreciation of Life, and Spiritual Change. Both the French and the original versions are valid and reliable for measuring post-traumatic growth^42,43^. In this study, Cronbach’s alpha is 0.96.

Finally, changes in domains of functioning were assessed using a homemade questionnaire (Changes in domains of functioning during pregnancy)^44^. Its 19 items are rated on a 5-point Likert scale, from 1 (*Greatly deteriorated*) to 5 (*Greatly improved*). The instrument yields three subscales: Self-Perception, Relationship with Partner, Relationship with Others. In this study, Cronbach’s alpha is 0.93.

### Data analytic strategy

Statistical analysis was performed using SPSS version 27. Baseline differences between treatment and control groups were assessed using independent samples *t* tests and chi-square tests. Differences between baseline and post-intervention on psychological distress, post-traumatic symptoms and positive affectivity were assessed using paired-sample *t* tests run separately for the Intervention and Control arms. Differences between the Intervention and Control groups on resilience-promoting factors were assessed using t-tests for independent samples. One-tailed significant tests were used given the a priori specified directional effects.

### Ethical approval

This study received ethical approval from the Comité d’éthique de la recherche du Centre intégré universitaire de santé et de services sociaux de la Mauricie-et-du-Centre-du-Québec (CER-2016-016) and the Comité d’éthique de la recherche avec des êtres humains de l’Université du Québec à Trois-Rivières (CER-16-226-10).

### Informed consent

Informed consent was obtained from all individual participants included in the study.

## Results

### Demographics

As a preliminary analysis, we compared participants who showed some interest in the intervention (n = 127) to participants who were not interested in participating in the program (n = 142) on baseline demographic and clinical data (see Fig. 1 and Table 1). Overall, participants who were not interested in the program were more frequently multiparous (n = 66, 46.5%) than women who showed interest in the intervention (n = 36, 28.6%), ${\chi }^{2}$(1) = 9.65, *p* = 0.002, and reported lower post-traumatic stress symptoms, *t*(248) = 2.22, *p* = 0.03. However, participants from both groups did not differ in terms of sociodemographic variables as well as baseline psychological distress and positive affectivity.

Women allocated to the Intervention and Control arms and who provided complete post-treatment follow-up were balanced on baseline sociodemographic variables, exposition to childhood interpersonal traumas and mental health symptoms (Table 2). Participants were mainly married or in common-law relationships with the other parent (n = 51, 98%), had some post-secondary education (n = 49, 92.4%) and were not part of a racial minority (n = 50 White/Caucasian, 96.2%). Median household annual income was around C$95 000 which represents a sufficient income for a family.

**Table 2:** Baseline participant characteristics of women who provided complete post data (n = 52).

Demographics	STEP-COVID (n = 17)	Control arm (n = 35)	Group differences (*p* value)^c^
Age, mean (SD)	30.06 (4.37)	30.34 (3.83)	0.82
Primiparous, n (%)	13 (76.5%)	26 (74.3%)	0.86
Gestational weeks, mean (SD)			
Baseline assessment	19.9 (6.65)	17.3 (3.21)	0.14
Post-intervention follow-up	36.22 (6.53)	38.0 (1.65)	0.27
Marital status, n (%)			
In relationship	17 (100%)	34 (97.1%)	0.82
Single	–	1 (2.9%)	
Education level, n (%)			
High school diploma or less	1 (5.9%)	2 (5.7%)	0.81
Collegial or professional training	8 (47.1%)	11 (31.4%)	
University degree	8 (47.1%)	22 (62.9%)	
Ethnicity, n (%)			
White	15 (88.2%)	35 (100%)	0.12
Minority	2 (11.8%)	–	
Annual household income, n (%)			
Can $34,999 or less	–	1 (2.9%)	0.16
Can $35,000–64,999$	–	8 (22.9%)	
Can $65,000$–94,999$	7 (41.2%)	9 (25.7%)	
Can $95,000 or more	10 (58.8%)	17 (48.6%)	
Childhood trauma, n (%)	2 (12.5%)	8 (23.5%)	0.36
Mental health, mean (SD)			
Psychological distress	19.71 (5.10)	17.83 (4.99)	0.21
Post-traumatic stress symptoms	11.0 (8.28)	11.29 (10.26)	0.92
Positive affects	31.82 (4.26)	33.46 (5.22)	0.27

^c^Two-sided *p*-values were obtained from *t*-tests for continuous variables and Chi-square tests for categorical variables.

### Primary outcomes

**Figure 2:**
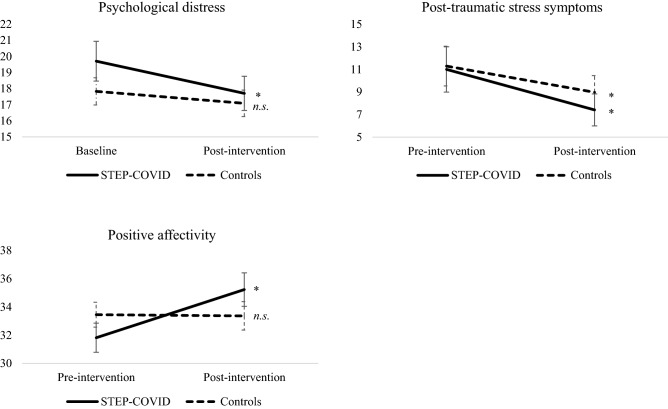
Change in mental health symptoms between baseline and post-intervention assessments. *Note* Length of the error bars represent the standard error for the mean. Means, SD and *t* tests are reported in Table S1. * *p* < 0.05; *n.s.* = non-significant.

As shown in Fig. 2, women in the Intervention arm showed a significant decrease in psychological distress, *t*(16) = 1.99, *p* = 0.03, *d* = 0.48, and a significant increase in positive affectivity, *t*(16) = − 3.37, *p* = 0.002, *d* = 0.82, before and after the STEP-COVID intervention, whereas women in the Control arm did not show such improvements [psychological distress, *t*(33) = 1.20, *p* = 0.10, *d* = 0.22; positive affectivity, *t*(33) = 0.09, *p* = 0.46, *d* = 0.01]. Women in both groups showed a significant decrease in post-traumatic stress symptoms [Intervention arm, *t*(16) = 2.16, *p* = 0.02; Control arm, *t*(33) = 1.979, *p* = 0.02] but a larger effect size was observed in the Intervention arm than in the Control arm (*d* = 0.52 and 0.34 respectively).

As shown in Table 3, women who completed the STEP-COVID program observed greater changes during pregnancy on resilience-promoting factors than non-participating women. More precisely, they reported feeling more capable of overcoming difficulties (*d* = 0.68), witnessing positive changes in their self-perception (*d* = 0.63) and observing positive changes in their relationship with their partner (*d* = 0.74). Participation in STEP-COVID was not associated with a greater appreciation of life, a greater appreciation of social support, the discovery of new life opportunities, or better relationships with significant others (excluding the partner).

**Table 3:** Perception of changes in resilience-promoting factors at the post-intervention assessment.

Variables	Intervention arm (n = 17) *M* (SD)	Control arm (n = 35) *M* (SD)	*t* (*df*)	*p* value	*d*
Post-traumatic growth					
Appreciation of life	9.00 (3.25)	8.30 (3.22)	− 0.84 (50)	0.20	0.25
Relating to others	17.18 (7.92)	15.03 (8.60)	− 0.87 (50)	0.19	0.26
New possibilities	10.65 (5.04)	9.26 (5.76)	− 0.85 (50)	0.20	0.26
Personal strength	10.65 (3.86)	7.63 (4.67)	− 2.31	0.01	0.68
Changes in functioning^a^					
Self-perception	7.44 (5.19)	4.62 (4.09)	− 2.08 (48)	0.02	0.63
Relationship with partner	5.81 (3.29)	3.38 (3.33)	− 2.40 (46)	0.01	0.74
Relationship with others	2.57 (2.62)	2.85 (2.36)	0.35 (39)	0.37	− 0.11

^a^Items used to assess changes in functioning are provided in Supp Methods (see the electronic supplement).

## Discussion

The COVID-19 pandemic has intensified the need for rapid research and novel clinical interventions^21^. This is particularly true for pregnant women, given the well-documented effects of prenatal distress on maternal functioning and offspring development^13,18–20^. However, intervention research is slow^45–47^ and rare are the empirically-supported interventions to buffer the deleterious impact of the COVID-19 pandemic on the mental health of pregnant women worldwide. Our findings that a brief online intervention could contribute to mitigating psychological distress and post-traumatic symptoms, increasing positive affectivity, enhancing resilience, improving the quality of the relationship with the partner and supporting a more positive view of self in pregnant women during the COVID-19 pandemic has important implications for clinical practice and public health. The results also offer additional support concerning the acceptability of STEP programs^48,49^ and the effectiveness of mentalization-based interventions during the prenatal period^22,50–52^.

The results of the study need to be considered in the light of some limitations. First, the sample size was small and participants were not assigned randomly to either the intervention or control conditions. However, conducting an RCT with a large sample size would take significant time and, as recently argued by Venta et al. (p. 202): “If the typical stages of intervention development or adaptation are undertaken prior to making efforts to support infants born during the COVID-19 pandemic and their mothers, we will be too late, missing the most plastic period of child development and one of the most vulnerable periods of a mother’s life”^21^. Another limitation is that the program is relatively brief and probably did not meet the needs of all participants. Further research, using mixed-methods and a larger sample of women participating in STEP-COVID should evaluate whether some participants might not improve in the course of the program, investigate the characteristics of these poor responders and clarify their unmet needs. Finally, the exclusive reliance on self-reported measures is another limitation and further studies should use structured clinical interviews, incorporate biological measures (e.g., inflammation) and evaluate whether the intervention contributes to mitigating the recently documented effect of prenatal stress during the COVID-19 pandemic on infant development^16^.

## Conclusion

Our findings have immediate implications for clinical practice. First, around half of the pregnant women who were approached during the recruitment process showed interest in participating in the program (see Fig. 1). This suggests that implementing and making accessible such psychological interventions on a large scale would successfully reach many pregnant women from the community and would respond to a definite need in this population. Second, the STEP-COVID program appears as an interesting avenue for a large-scale deployment. Indeed, the program is relatively brief (six-sessions), manualized, designed to be offered online, and the current pilot data support its effectiveness.

## Supplementary Information

Supplementary Information.

## Data Availability

The datasets used and/or analyzed during the current study are available from the corresponding author on reasonable request.
