# Supplementary material for: STEP-COVID: a pilot study of a prenatal intervention for pregnant women during the COVID-19 pandemic
Source: Sci Rep. 2023 Apr 20;13:6466. doi: 10.1038/s41598-023-33369-8 (PMC10116444; doi:10.1038/s41598-023-33369-8)
Supplement: Supplementary file 1 — Supplementary Information. [file 41598_2023_33369_MOESM1_ESM.docx]

**SUPPLEMENTAL METHODS:**

**Assessment of changes in domains of functioning during pregnancy**

The following questionnaire concerns changes that may have occurred during pregnancy in different areas of your life. For each of the statements below, please indicate whether you have observed any changes and whether these changes were positive or negative.

(1) It has greatly deteriorated

(2) It has deteriorated a little

(3) I did not notice any change

(4) It has slightly improved

(5) It has greatly improved

| **Since the beginning of the pregnancy, I have noticed changes in...** | **Greatly deteriorated** | Deteriorated a little | **No change** | Slightly improved | **Greatly improved** |
| --- | --- | --- | --- | --- | --- |
| 1. My level of satisfaction in my relationship with my partner | 1 | 2 | 3 | 4 | 5 |
| 1. The way I communicate with my partner | 1 | 2 | 3 | 4 | 5 |
| 1. My ability to confide in my partner | 1 | 2 | 3 | 4 | 5 |
| 1. My interest in my partner's feelings | 1 | 2 | 3 | 4 | 5 |
| 1. My ability to provide my partner with emotional support | 1 | 2 | 3 | 4 | 5 |
| 1. My self-esteem | 1 | 2 | 3 | 4 | 5 |
| 1. My sense of being able to deal with the challenges I face | 1 | 2 | 3 | 4 | 5 |
| 1. My psychological well-being | 1 | 2 | 3 | 4 | 5 |
| 1. My ability to manage stress and difficult emotions | 1 | 2 | 3 | 4 | 5 |
| 1. My ability to recognize my needs | 1 | 2 | 3 | 4 | 5 |
| **Since the beginning of the pregnancy, I have noticed changes in...** | **Greatly deteriorated** | Deteriorated a little | **No change** | Slightly improved | **Greatly improved** |
| 1. My ability to assert myself | 1 | 2 | 3 | 4 | 5 |
| 1. My feelings relating to my past | 1 | 2 | 3 | 4 | 5 |
| 1. My confidence in parenting | 1 | 2 | 3 | 4 | 5 |
| 1. My relationship with my mother | 1 | 2 | 3 | 4 | 5 |
| 1. My relationship with my father | 1 | 2 | 3 | 4 | 5 |
| 1. My relationship with my sibling(s) | 1 | 2 | 3 | 4 | 5 |
| 1. My relationship with my other child(ren) | 1 | 2 | 3 | 4 | 5 |
| 1. My ability to confide in my loved ones. | 1 | 2 | 3 | 4 | 5 |
| 1. My ability to ask for help or support when I need it | 1 | 2 | 3 | 4 | 5 |

**Table S1.** Change in mental health symptoms between baseline and post-intervention assessments

| Variables | **STEP-COVID (Intervention arm)** | | | | | **Control arm** | | | | |
| --- | --- | --- | --- | --- | --- | --- | --- | --- | --- | --- |
|  | Baseline  *M* (SD) | Post-intervention  *M* (SD) | *t* (16) | *p* value | *d* | Baseline  *M* (SD) | Post-intervention  *M* (SD) | *t*(33) | *p* value | *d* |
| Psychological distress | 19.71 (1.24) | 17.71 (1.06) | 1.99 | .03 | 0.48 | 17.83 (0.85) | 17.09 (0.82) | 1.29 | .10 | 0.22 |
| Post-traumatic stress symptoms | 11.0 (2.01) | 7.41 (1.41) | 2.16 | .02 | 0.52 | 11.29 (1.76) | 8.97 (1.48) | 1.98 | .02 | 0.34 |
| Positive affectivity | 31.82 (1.03) | 35.24 (1.18) | -3.37 | .002 | 0.82 | 33.46 (0.88) | 33.37 (1.01) | 0.09 | .46 | 0.01 |

*Note.* One-sided *p*-values were obtained from *t*-tests.
